# Trends of Bacterial Keratitis Culture Isolates in Jerusalem; a 13- Years Analysis

**DOI:** 10.1371/journal.pone.0165223

**Published:** 2016-11-28

**Authors:** Michael Politis, Denise Wajnsztajn, Boris Rosin, Colin Block, Abraham Solomon

**Affiliations:** 1 Department of Ophthalmology, Hadassah-Hebrew University Medical Center, Jerusalem, Israel; 2 Department of Clinical Microbiology, Hadassah-Hebrew University Medical Center, Jerusalem, Israel; Kent State University, UNITED STATES

## Abstract

**Purpose:**

To describe the trends in pathogens and antibacterial resistance of corneal culture isolates in infectious keratitis during a period of 13 years at Hadassah-Hebrew University Medical Center.

**Methods:**

A Retrospective analysis of bacterial corneal isolates was performed during the months of January 2002 to December 2014 at Hadassah Hebrew University Medical Center. Demographics, microbiological data and antibiotic resistance and sensitivity were collected.

**Results:**

A total of 943 corneal isolates were analyzed during a 13 year period. A total of 415 positive bacterial cultures and 37 positive fungal cultures were recovered, representing 48% of the total cultures. The Annual incidence was 34.78 ± 6.54 cases. The most common isolate was *coagulase-negative staphylococcus* (32%), which had a significant decrease in trend throughout the study period (APC = -8.1, p = 0.002). Methicillin-resistant Staphylococcus aureus (MRSA) appears to have a decrease trend (APC = -31.2, P = 0.5). There was an increase in the resistance trend of *coagulase-negative staphylococci* to penicillin (APC = 5.0, P = <0.001). None of the pathogens had developed any resistance to Vancomycin. (P = 0.88).

**Conclusions:**

Coagulase negative staphylococci were the predominant bacteria isolated from patients with keratitis. There was no significant change in the annual incidence of cases of bacterial keratitis seen over the past 13 years. Keratitis caused by MRSA appeared to decrease in contrast to the reported literature.

## Introduction

Bacterial keratitis is a significant cause of visual loss. A targeted therapy based on corneal cultures and sensitivity of antibiotics is essential for the effective management of bacterial keratitis[1]. While awaiting cultures, empiric treatment should be started immediately and the antibiotic chosen should be of sufficiently broad spectrum to cover likely pathogens based on local bacterial prevalence and antibiotic susceptibilities.[2] Since regional differences exist in the etiologies of bacterial keratitis[3,4], good local epidemiological data are needed for better empirical treatment of bacterial keratitis.

Many community-based ophthalmologists elect to treat bacterial keratitis with broad-spectrum antibiotics without corneal scraping and susceptibility results.[5] In our institution we routinely culture all cases of suspected bacterial keratitis and start combination therapy with topical fortified cefazolin and gentamicin.

Recent shifts in bacterial etiologies have been reported where an increase in methicillin resistant Staphylococcus aureus has been observed [6,7]. Other trends in antibiotic susceptibility are changing depending on the way bacterial keratitis is treated, especially the recent emergence of resistance to fluoroquinolones among common corneal pathogens. [8–11]

The aim of this study was to describe the epidemiology of bacterial keratitis culture isolates and the trends in etiology and antibacterial resistance during the past 13 years at Hadassah-Hebrew University Medical Center.

## Methods

This study conformed to the provisions of the declaration of Helsinki and was approved by the Institutional Review Board of the Hadassah-Hebrew University Medical Center (HMO-0664-13). The Institutional Review Board waived the need for informed consent from all patients who had been cultured for microbial keratitis during the study period, and their culture samples data was included in this study.

### Data collection

This is a retrospective study; records were collected prospectively from January 2002 to December 2014. After collecting the sample it was sent immediately to the microbiology department and registered by the IT section recording all corneal samples including culture results, demographics, time of corneal sampling, type of sample, identity of the organism and antibiotic susceptibility. Special care was taken to include samples collected only by corneal scraping and to ensure that only the first isolation of a particular organism was counted for each patient. We encountered 32 cases of mixed infections that were included in the positive culture rate calculations. However, they were excluded out of the trend analysis.

### Corneal culture sample

Microbiological examinations were conducted in the Clinical Microbiology Laboratory of the Hadassah-Hebrew University Medical Center. Corneal cultures were taken at the ER/clinic, using a sterilized Kimura spatula, following a diagnosis of suspected microbial corneal ulcer, after no antibiotic treatment was given or following a 12 hours therapeutic window. Topical anesthetic drops were used and special care was taken not to touch the lid margin or lashes to avoid contamination. After collecting the sample it was plated by an Ophthalmology resident directly onto 5% sheep blood agar and chocolate agar (Novamed, Jerusalem) using conventional “C” inocula[12], and two smears were prepared on clean microscope slides for Gram stain and additional staining methods as necessary. All corneal specimens submitted to the laboratory were routinely tested for fungal pathogens as well. This included microscopic evaluation using calcofluor white fluorescent staining and culture on Sabouraud Dextrose Agar and Brain-Heart Infusion Agar. Nevertheless, this report does not include detailed fungal analysis.

Organisms were identified using routine laboratory methods with conventional phenotyping, being largely replaced, from 2013, by Matrix-assisted laser desorption-ionization-time of flight (MALDI-TOF) mass spectrometry (VITEK MS, Biomerieux, France). This technique significantly improved the laboratory’s ability to identify some genera to the species level.[13] Bacterial susceptibility to antibiotics was assessed by the agar disc-diffusion method using CLSI methods and criteria (Clinical Laboratory Standards Institute, USA, as annually updated).

### Data analysis

The study was divided into 3 periods for analysis: January 2002 to December 2005, January 2006 to December 2009 and January 2010 to December 2014. To evaluate the effect of time on the day of culture, we recorded the time of the day of the corneal sample and divided into periods following the regular 8 hour work period: from 8:00am to 3:00pm, 3:01pm to 11:00pm and 11:01pm to 7:59am.

### Statistics

The statistical analysis was made using SPSS software version 22 (SPSS Inc, Chicago, IL). Descriptive statistics were used to describe the sample. Joinpoint regression analysis program version 4.2.0.2 was used to determine trends. The Joinpoint program selects the best fitting piece-wise continuous log- linear model. The permutation test was performed to determine the minimum number of “joinpoints” necessary to fit the data [14]. For the study period, we utilized trend analysis for selected antibiotic resistance and sensitivity trends. Trends were also assessed for selected pathogens and displayed in tables using the annual percent change (APC) with 95% confidence intervals.

Unpaired t-test was used to be able to evaluate if the introduction of Matrix-assisted laser desorption-ionization-time of flight (MALDI-TOF) mass spectrometry improved the yield of positive culture results.

Binomial proportion testing was used to prove significance in corneal sample culture collection and the yield of positive corneal isolates.

## Results

**Fig 1:**
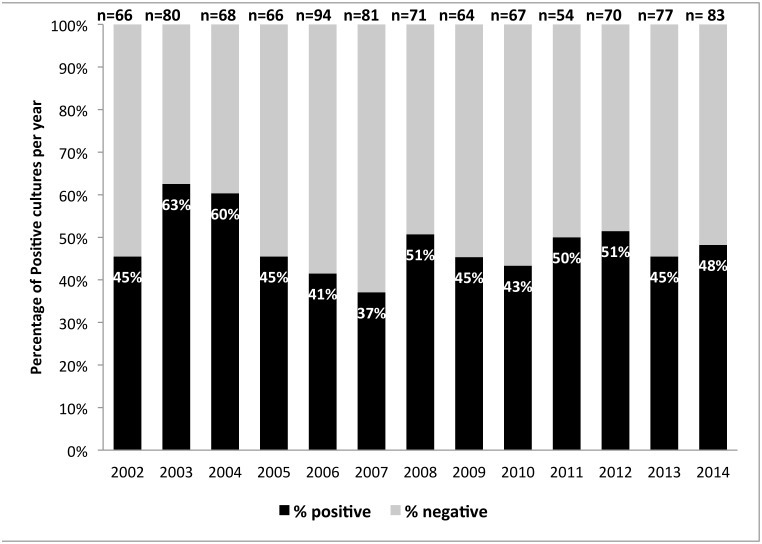
Bacterial keratitis culture distribution per year 2002–2014.

During the 13-year study period (2002–2014) a total of 943 corneal scrapings were sent for cultures. A total of 415 positive bacterial cultures and 37 positive fungal cultures were recovered, representing 48% of the total cultures. Bacterial keratitis represented 92% out of the total positive cultures. 53% of the patients were male. The mean age of patients with positive bacterial cultures was 46.96 ± 25.16 years. The mean number of positive bacterial corneal scrapings per year was 34.78 ± 6.54 cases. Fig 1 (S1 Table) shows the proportions of positive and negative cultures for each of the study years separately. The proportion of positive cultures was essentially stable despite an apparent minor decrease, APC = -1.6 (P = 0.3).

There was no significant difference when comparing the yield of positive culture rate and identification of organisms, before and after the introduction of Matrix-assisted laser desorption-ionization-time of flight (MALDI-TOF) mass spectrometry (VITEK MS, Biomerieux, France) (P = 0.47).

### Changes in bacterial trends

**Fig 2:**
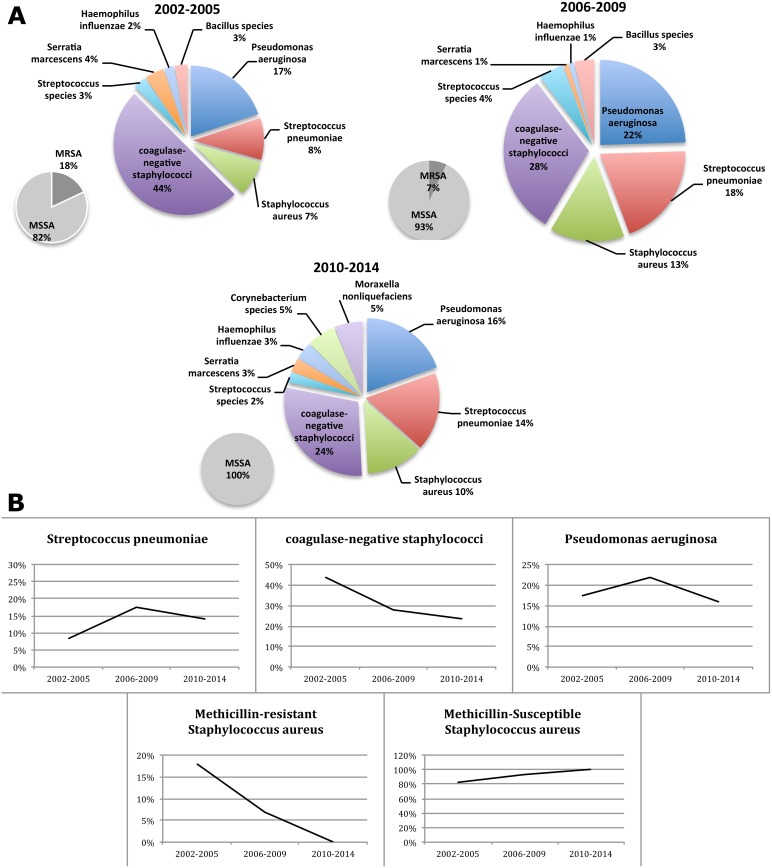
(A) Shifting proportions of bacterial keratitis isolates by year period 2002–2014. (B) Trends of bacterial keratitis isolates by year period 2002–2014.

Shifts in the proportions of the main etiological agents of bacterial keratitis were detected during the study (Fig 2A and 2B) (S1 Table). During the period of 2002–2005: Coagulase-negative staphylococci, Pseudomonas aeruginosa, Streptococcus pneumoniae and Staphylococcus aureus comprised 44%, 17%, 8% and 7% respectively of the total positive cultures in the mentioned study period. For the period of 2006–2009: Coagulase-negative staphylococci decreased to 28%, Pseudomonas aeruginosa increased to 22%, Streptococcus pneumoniae increased to 18% and Staphylococcus aureus increased to 13%. For the period of 2010–2014: Coagulase-negative staphylococci remained low at 24%, Pseudomonas aeruginosa dropped to 16%, Streptococcus pneumoniae to 14% and Staphylococcus aureus to 10%. These four groups thus represented 76%, 81% and 64% of all bacterial isolates in the three periods. The drop in laboratory isolation of coagulase negative staphylococci explains the change in trends among the four groups of bacterial cultures. This also emphasizes the need to scrutinize the taxa that had greater proportional (and perhaps numerical) representation in the 2010–2014 periods.

### Trend analysis

In order to evaluate the changes in positive bacterial corneal scrapings and their resistance to common antibiotics during the study period, we performed trend analysis. This analysis determines the Annual Percent Change (APC) and assigns statistical significance to the slope.

The only statistically significant trend was seen for Coagulase-negative staphylococci with an Annual Percent Change (APC) of -8.1 (P = 0.002). The apparent positive APC trends for Streptococcus pneumoniae, methicillin-sensitive Staphylococcus aureus (MSSA) and Escherichia coli were not significant, being +7.4 (P = 0.2), +7.5 (P = 0.2) and +14.7 (P = 0.1) respectively. Methicillin-resistant Staphylococcus aureus (MRSA), Pseudomonas aeruginosa, Haemophilus influenzae and Streptococcus species had a non-statistical significant decrease in trend APC of– 31.2 (P = 0.5), -5.3 (P = 0.1), -8.3 (P = 0.1) and -3.5 (P = 0.5) respectively (Table 1) (S1 Table).

**Table 1:** Trend analysis of bacterial keratitis isolates by year period 2002–2014.

Common bacterial isolate	2002–2005	2006–2009	2010–2014	APC*	P value
Grand Total	155	119	157	-1.6	0.3
Coagulase-negative staphylococci	68	33	37	-8.1	0.002
Pseudomonas aeruginosa	27	26	25	-5.3	0.1
Streptococcus pneumoniae	13	21	22	7.4	0.2
Staphylococcus aureus	11	15	16	-0.8	0.9
MRSA	2	1	0	-31.2	0.5
MSSA	9	14	16	7.5	0.2
Haemophilus influenzae	4	3	2	-8.3	0.1
Streptococcus species	4	5	3	-3.5	0.5
Escherichia coli	1	1	3	14.7	0.1

*APC: Annual Percent Change

### Common antibiotic resistance trends

Coagulase-negative Staphylococci showed a statistical significant trend with an increase in the proportion resistant to penicillin in the study period (APC = 5.0, P = <0.001).

The antibiotics analyzed had no statistically significant trend, no vancomycin resistance was observed throughout the 3 study periods for coagulase-negative Staphylococci, Staphylococcus aureus and Streptococcus pneumoniae (Table 2) (S1 Table).

**Table 2:** Trend analysis of antibiotic resistance for common pathogens.

		2002–2005		2006–2009		2010–2014		APC*	P value
		%	N	%	N	%	N		
**Pseudomonas aeruginosa**	Amikacin	0%	0	0%	0	0%	0	0.00	>0.05
	Cetazidime	0%	0	0%	0	0%	0	0.00	>0.05
	Ciprofloxacin	0%	0	8%	2	0%	0	0.00	>0.05
	Gentamicin	7%	2	12%	3	0%	0	-31.20	0.4
**Staphylococcus aureus**	Chloramphenicol	18%	2	7%	1	0%	0	-31.20	0.2
	Ciprofloxacin	0%	0	0%	0	0%	0	0.00	>0.05
	Gentamicin	9%	1	7%	1	6%	1	0.00	>0.05
	Oxacilin/methicilin	18%	2	7%	1	0%	0	-31.20	0.2
	Penicilin	9%	1	13%	2	6%	1	0.00	>0.05
	Vancomycin	0%	0	0%	0	0%	0	0.00	>0.05
**Coagulase-negative staphylococci**	Chloramphenicol	13%	9	27%	9	5%	2	-17.10	0.3
	Ciprofloxacin	0%	0	6%	2	14%	5	63.10	0.2
	Gentamicin	13%	9	9%	3	16%	6	-4.90	0.8
	Penicilin	28%	19	70%	23	76%	28	5.00	<0.001
	Vancomycin	0%	0	0%	0	0%	0	0.00	>0.05
**Streptococcus pneumoniae**	Ceftriaxone	0%	0	0%	0	0%	0	0.00	>0.05
	Chloramphenicol	0%	0	0%	0	0%	0	0.00	>0.05
	Penicilin	8%	1	43%	9	50%	11	35.00	0.3
	Vancomycin	0%	0	0%	0	0%	0	0.00	>0.05
**Total**	Cefazolin	8%	12	4%	5	4%	7	-6.50	0.6
	Gentamycin	8%	12	6%	7	6%	10	-2.30	0.8
	Vancomycin	0%	0	0%	0	0%	0	0.00	>0.05
	Ciprofloxacin	0%	0	3%	4	5%	8	72.90	0.2

*APC: Annual Percent Change

### Timing of corneal cultures and results

**Fig 3:**
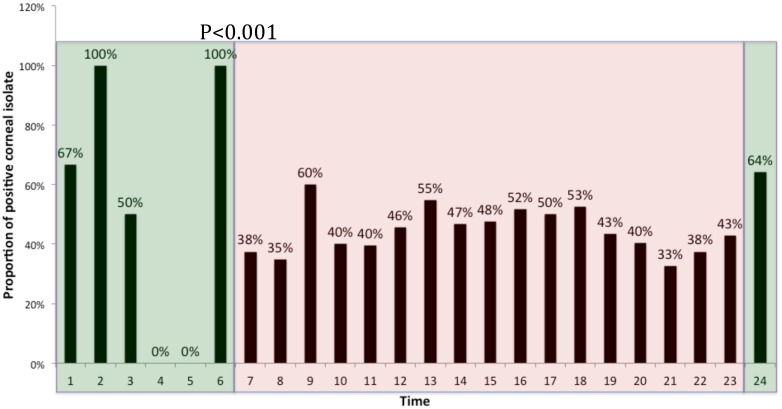
Proportion of Positives Corneal Isolates in a 24-hour period during 2002–2014.

The average hourly yield of positive cultures for bacterial keratitis has been proven to be significantly lower during the night (23:00–06:00) compared to day (07:00–15:00) and evening (16:00–22:00) shifts P<0.01 (Fig 3) (S1 Table).

## Discussion

Bacterial keratitis remained the most common cause of infectious keratitis in our hospital. Coagulase negative staphylococci were the predominant bacteria isolated from patients with keratitis. An increased trend in Penicilin resistance to coagulase-negative staphylococci was demonstrated. There was no significant change in the overall number of cases of bacterial keratitis seen over the past 13 years.

This study is the first to bring data on bacterial keratitis culture isolates from a major tertiary medical center in Israel and with a relatively large follow up period compared to most of the studies commonly cited in the literature (Table 3).

**Table 3:** Literature review summary table.

Author	Year published	Follow-up (Years)	Location	Number of corneal scrapes	Comments
Alexandrakis G et al.	2000	9	Florida	2920	Increase in number of S. aureus keratitis in the study period.
					Decrease in the number of Pseudomonas aeruginosa keratitis in the study period
					Increasing laboratory resistance of S. Aureus keratitis isolates to fluoroquinolones
Lalitha et al.	2014	11	India	5912	Annual number of keratitis cases due to bacteria decrease and the annual number due to fungus increased
Lichtinger et al.	2012	11	Toronto	1701	Decrease trend in Gram-positive isolates
					Most common isolate overall was coagulase-negative Staphylococcus
					Increase trend toward laboratory resistance to methicillin
					MRSA was resistant to cefazolin and sensitive to vancomycin in all isolates
Sand et al.	2015	4	Los Angeles	290	Coagulase negative Staphylococcus most common gram-positive isolate, Pseudomonas aeruginosa most common gram-negative isolate
					Ciprofloxacin and levofloxacin was susceptible on all tested pathogens in 73% and 81% respectively
Shalchi Z et al.	2011	10	UK	476	Increase in the number of gram-negative isolates in the study period
					In vitro wide spread gram-negative resistance to chloramphenicol
					No increase trend found on ciprofloxacin resistance
					97.3% of isolates were sensitive to combination of gentamicin and cefuroxime
Zhang et al.	2008	4	China	279	Pseudomonas sp. most common organism isolated
					Ciprofloxacin showed the highest rate of resistance in all isolates
					The resistance of isolates from patients older than 60Y to ciprofloxacin, levofloxacin and tobramycin was higher that from younger adults (14–59)
Hong J et al.	2013	6	Shanghai	436	Most common isolate Streptococcus species
					MRSA was found in 8.3% of the S. Aureus isolates
					Increase trend toward laboratory resistance to fluoroquinolones
					No resistance found for Gram-positive isolates to vancomycin
Hernandez-Camarena JC et al.	2015	10	Mexico city	616	Most common isolated pathogen was Staphylococcus epidermidis
					Non-significant increasing trend on Gram-negative isolates
					MRSA was present in 45% out of the total S. Aurues isolates
					Increasing resistance to ceftazidime for Pseudomonas aeruginosa
Lavinsky F et al.	2013	3	Tel Aviv, Israel	276	Staphylococcus aureus was the most common isolate found on corneal scrapings
Orlans HO et al.	2011	10	UK	467	Most common isolate Staphylococci sp.
					Increase in the number of Coagulase-negative Staphylococci isolates
					Increase resistance of non-Pseudomonas Gram-negative isolates to chloramphenicol
					93.2% of all isolates were susceptible to ciprofloxacin and 99.5% to either gentamicin or cefuroxime
Ng AL-K et al.	2015	10	Hong Kong	347	Most common isolate overall was coagulase-negative Staphylococcus
					Fluoroquinolone was found susceptible in 93.6% of al Gram-negative and in 100% of all Pseudomonas aeruginosa
					No emergence of resistant strain during the study period
Ibrahim MM et al.	2011	3	Brazil	118	Predominant bacterial pathogen isolated was S. Epidermidis
Chang VS et al.	2015	20	Pittsburgh,USA	398	Analyzed only antibiotic susceptibility to MRSA and MSSA keratitis
					MRSA represented 30.7% of total S. aureus isolates
					Vancomycin was susceptible to all S. aureus
					MRSA was found more resistant to second-generation fluoroquinolones than to the fourth-generation fluoroquinolones
					Increase in resistance to fourth-generation fluoroquinolone was detected during the study period for MSSA and MRSA
Politis et al.		13	Jerusalem,Israel	943	Cultures were recovered in 48% of all cultures
					Most common isolate overall was coagulase-negative Staphylococcus
					Significant decrease trend in cases of coagulase-negative Staphylococcus (APC -8.1)
					Increase trend of coagulase-negative Staphylococcus resistance to penicillin.
					Vancomycin was susceptible to all pathogens
					Inverted correlation between temperature and number of cases of bacterial keratitis

Bacterial keratitis is a devastating blinding disease. Appropriate therapy is crucial for eradication of the infective organism. [4] Bacterial resistance has been a concern since the 1950´s when Australia and Great Britain first reported penicillin resistance in Staphylococcus aureus.[15] Geographical location and cultural features of study populations are factors that are related to the differences in bacterial resistance patterns between countries.[16] Up to now there has been a dearth of reliable and comprehensive data regarding the etiology of bacterial keratitis in Israel.

In our 13 year study, 943 corneal scrapings were performed, with a mean of 34.78 ± 6.54 positive bacterial cultures per year. There was a stable trend of total bacterial cultures during the study period, contrary to the study by Lichtinger et al. [4] who had found a decreased trend of positive bacterial cultures during his study period, specifically of gram positive bacteria. However, this could reflect the facts that community based ophthalmologists are treating many patients empirically, and successfully, so that most patients are not referred to tertiary care centers.

**Fig 4:**
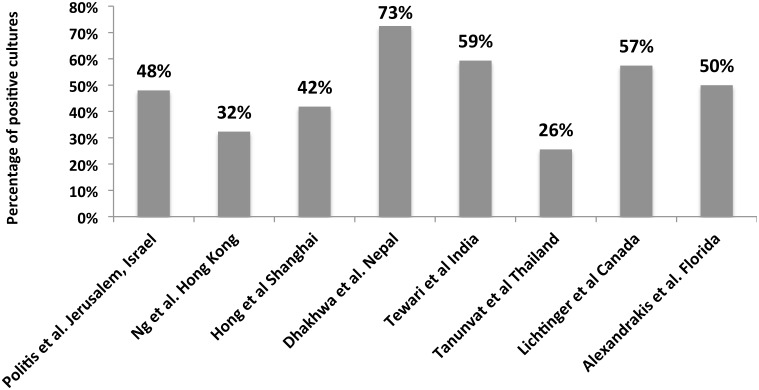
Comparison of positive culture isolates in the published literature.

Our positive culture rate was 48%, which is consistent with the range reported in the literature of 36–86%[1,4,7,17–19] (Fig 4). Bacterial isolates represented 92% of the total cultures as seen in other series where bacteria account for 88–91% [4,17] of positive cultures.

The most common bacterial isolates throughout the study period were coagulase negative staphylococci, in accordance with many studies[4,17,20,21] including an Israeli report by Mezer et al[22]. However, a study by Lavinsky et al[18] showed that Staphylococcus aureus was the most common pathogen. We found a significant decreasing trend in the proportion of coagulase negative staphylococci in corneal cultures during the study period. This may have been related to increases in the relative frequencies of other pathogens like Pseudomonas aeruginosa, Staphylococcus aureus and Streptococcus pneumonia.

Fungal keratitis represented 8.2% (N = 37) of the total positive cultures, consistent with other reports where the proportions ranged from 5–34%[3,17,23].

Methicillin resistant Staphylococcus aureus has become increasingly important in the management of bacterial keratitis due to the challenges in treatment choices that this represents. Community acquired MRSA infections have become a significant problem in Israel [24]. In our study the percentage of MRSA isolates decreased from 18% in the first study period of 2002–2005 to 0% in the last study period of 2010–2014 with a non-statistical decrease in trend. This differs significantly from frequencies reported in the literature [4,6,7,17,25]. Furthermore, much higher frequencies of MRSA isolates from corneal cultures have been reported in other studies, ranging from 42–45%[6,7]. The choice of treatment for culture confirmed MRSA keratitis has been even more challenging where some studies have found an increase in resistance to fluoroquinolones, thus resulting in a shift to fortified topical vancomycin[26,27]. In our study we found a 0% resistance rate to vancomycin supporting the choice of vancomycin as a preferred treatment for culture confirmed MRSA keratitis. However, despite reports[17] of resistance to vancomycin, this agent remains a suitable option for treatment in our population.

In an attempt to identify factors that might influence the recovery of bacteria from corneal cultures, we analyzed the percentage of positive and negative cultures by time of sample collection. We hypothesized that the time of day and the experience of the individual taking the specimen might be associated with pathogen recovery rates. In our Ophthalmology Department, residents work together with fellows and senior Ophthalmologists during the day in the clinics from 07:00 to 15:00. After 15:00 only the residents are on call, with limited support from senior personnel. The proportion of positive cultures decreased significantly when the sample was taken after 23:00. This phenomenon might be explained by the stressful workload in the evening and the lack of experienced senior ophthalmologist, resulting in suboptimal technique in culture recollection. However, other factors should be considered, such as delays in transportation of specimens to the laboratory and staff availability for processing them.

We recognize that our study had several limitations. Most of the community based empirical treatment of corneal ulcers are based on the fourth generation fluorquinolones [28]. These drugs were not available in our hospital formulary, and therefore were not included in the microbiology laboratory susceptibility analysis during the study period. In addition, our study was conducted in a university referral center serving as a tertiary care hospital; therefore our findings may not be generalizable to other settings or populations. The data presented in our study represents the culture isolates found in a patient population, which is referred by community Ophthalmologists for management in a tertiary referral center, and therefore may include pathogens and susceptibility trends which are different than those found in corneal infections treated in the community.

This study has provided the first trend analysis of bacterial keratitis in Israel. Coagulase negative staphylococci were the predominant bacteria isolated from patients with keratitis, with a stable trend of positive cultures of bacterial keratitis in the past 13 years. Based on this study, we suggest the use of routine culture techniques for each case with a suspected infectious keratitis, with the regular use of antibiotics sensitivity and resistance in deciding the treatment plan. In addition, we recommend using fortified topical vancomycin as part of the regimen as a first-line agent in culture-confirmed MRSA, which is non-sensitive to the regular empiric treatment with cefazolin and gentamicin.

Continued monitoring of the microbial causes and their antibiotic susceptibilities of keratitis should be established. The policy of testing only drugs available in the hospital formulary should be reconsidered. This would allow the accumulation of much needed data regarding community-associated pathogens as well as to provide early warning of the emergence of new resistant pathogens.

## Supporting Information

S1 Table Dataset table demographics and antibiotic resistance. (XLSX)
